# Regulation of the *Xenopus Xsox17α*_*1*_ promoter by co-operating VegT and Sox17 sites

**DOI:** 10.1016/j.ydbio.2007.07.028

**Published:** 2007-10-15

**Authors:** Laura Howard, Maria Rex, Debbie Clements, Hugh R. Woodland

**Affiliations:** Department of Biological Sciences, University of Warwick, Coventry, CV4 7AL, UK

**Keywords:** Sox17, VegT, Endoderm, *Xenopus*, Transgenic embryo, Promoter, TGF-β

## Abstract

The gene encoding the Sox F-group transcription factor Xsox17α_1_ is specifically expressed throughout the entire region of the *Xenopus* blastula fated to become endoderm, and is important in controlling endodermal development. *Xsox17α*_*1*_ is a direct target of the maternal endodermal determinant VegT and of Sox17 itself. We have analysed the promoter of the *Xenopus laevis Xsox17α*_*1*_ gene by transgenesis, and have identified two important control elements which reside about 9 kb upstream at the start of transcription. These elements individually drive transgenic endodermal expression in the blastula and gastrula. One contains functional, cooperating VegT and Sox-binding consensus sites. The Sox sites in this region are occupied in vivo. The other responds to TGF-β signals like Activin or Nodals that act through Smad2/3. We propose that these two regions co-operate in regulating the early endodermal expression of the *Xsox17α*_*1*_ gene.

## Introduction

The endoderm of *Xenopus* embryo arises in two successive phases, involving firstly cell-autonomous gene action, followed by dependence on cell signalling. The cell-autonomous phase is directly initiated by the maternal T-box transcription factor VegT, but then some key genes associated with endodermal differentiation become dependent on signalling by the group of TGF-β family members that signal through Smad2/3 These include the Nodal-related proteins or Xnrs, Derrière, Vg1 and Activin. In this second phase, cells are sensitised to TGF-β signalling by the maternal VegT that they inherit (Clements et al., 2001; Clements and Woodland, 2003; Engleka et al., 2001; Hudson et al., 1997; Yasuo and Lemaire, 1999). Since the essential TGF-β signalling molecules are themselves induced by VegT, and VegT is indispensable for endoderm development (Xanthos et al., 2001), the overall effect is that only a critical mass of VegT-containing cells can generate sufficient signalling to sustain expression of these key endodermal genes. Scattered VegT containing cells will fail to become endoderm and conform to their surroundings. In contrast, some other genes (e.g. *Xnr4*), which are directly induced by VegT do not become signal-dependent. Finally, in the gastrula, endodermal gene expression becomes independent of cell signalling (Yasuo and Lemaire, 1999). This interpretation of endoderm initiation, establishment and maintenance is heavily based on studying the expression of the VegT target *Xsox17*, an HMG-box transcription factor, although *Mix.1* and *Mixer* behave in a similar fashion.

There are three *Sox17* genes in *Xenopus laevis*, *Xsox17α*_*1*_, *α*_*2*_ and *β*, but since no differences in their activity have so far been detected, for most purposes we refer to them collectively as *Xsox17*. The transcription of these Sox F group genes is activated prior to the mid-blastula transition (MBT), when very low levels are found ubiquitously, but Xsox17 transcription in the vegetal pole is enormously upregulated at MBT, precisely marking out the territory of the future endoderm through late blastula, gastrula and neurula stages (Hudson et al., 1997; Zorn et al., 1999). The *Xsox17* genes have a key role in endoderm formation. Their ectopic expression induces endodermal gene expression, as well as changing the fate of cells. Interfering with their expression with a dominant negative Engrailed *Xsox17* fusion construct has the converse effect, inhibiting endodermal gene expression and shifting cells out of an endodermal fate in intact embryos (Clements and Woodland, 2000; Hudson et al., 1997). The use of antisense morpholino oligos shows that the individual genes have non-redundant late roles in the developing mid- and hindgut, but that together they are needed for the completion of gastrulation (Clements et al., 2003). This correlates well with the essential role of the single-murine *Sox17* gene for early development of endoderm fated to become mid- and hindgut, although in the absence of Sox17 there is also later loss of foregut cells (Kanai-Azuma et al., 2002). In zebrafish, two related Sox genes, *Casanova* and *Sox17*, are important in forming the endoderm; the former is more upstream and its mutants indicate that it has a vital role in endoderm development (Aoki et al., 2002). While mutations in the zebrafish *Sox17* gene have not been described, it is likely that in the zebrafish endodermal gene network the combined action of *Casanova* and *Xsox17* are roughly equivalent to the overall action of *Xsox17* in *Xenopus*, particularly allowing for the fact that *Casanova* expression in the yolk syncytial layer is Nodal-independent, allowing a parallel with the *Xenopus* cell autonomous phase to be drawn (Kikuchi et al., 2001; Woodland and Clements, 2003). In *Xenopus*, ablation of Xsox17 expression with morpholinos halts gastrulation at an early stage. However, the immediate effects on gene expression were initially reported to be modest and were restricted to the direct Xsox17 targets *Endodermin* and *Hnf-1β* (Clements et al., 2003). However, much wider effects have now been observed using microarrays (Sinner et al., 2006).

Since *Xsox17* is a crucial gene in the early endoderm, and its expression defines the endodermal territory, we have analysed the regulatory elements in the *Xsox17α*_*1*_ promoter approximately 9 kb upstream of transcriptional initiation. We have identified two small elements, which can confer endodermal expression on a reporter gene in the early embryo. We have analysed one of these in detail and show that its activity depends on co-operating VegT and Sox17-binding sites, whereas the other regulatory region responds to Activin.

## Materials and methods

### Biological materials

Eggs of *X. laevis* were obtained, fertilised, dejellied and cultured by standard methods, as described previously (Wilson et al., 1986). Oocytes, complete with their follicle, were manually removed using watchmakers forceps in modified Barths' saline (MBS; 110 mM NaCl, 1 mM KCl, 2.4 mM NaHCO_3_, 0.33 mM Ca(NO_3_)_2_, 0.41 mM CaCl_2_, 0.8 mM MgSO4, 15 mM Tris–HCl, pH 7.5).

### Transgenic methods

These followed the procedure of Kroll and Amaya (1996), except that a reduced amount of egg extract was used in the incubation of stored frozen sperm nuclei with DNA (2 μl in a 25 μl reaction). The reaction typically contained 150–200 ng of linearised plasmid DNA and 0.5 μl of a 1:200 dilution of restriction enzyme (2 units/ml). Injections of dejellied unfertilised eggs were performed in 6% Ficoll, 0.4× Marc's modified Ringer (MMR) or 0.4× MBS in polyheme-coated plastic dishes. Correctly cleaving eggs were sorted at the 2- to 4-cell stage and incubated in 6% Ficoll, 0.1× modified Barth's medium. GFP fluorescence was monitored using a Leica MZFLIII dissecting microscope.

### Transient transgenesis in embryos and oocytes

For transient expression in embryos, DNA constructs were linearised and purified with the Qiagen gel extraction kit. 50 pg DNA together with 5 pg control *Renilla* luciferase reporter in 10 nl water were injected bilaterally, with or without mRNA, into the animal or vegetal poles of 2-cell embryos, cultured in 6% Ficoll, 0.4× MBS. Embryos were analysed at gastrula or neurula stages using the Promega Dual Luciferase Reporter Assay System. Triplicate batches of 10 embryos were homogenised in 600 μl passive lysis buffer and incubated on ice for 10 min. Samples were centrifuged at 13,000 rpm for 2 min at 4 °C, and supernatants removed. They were equilibrated to room temperature for 30 min, and 60 μl assayed for bioluminescence after addition of 50 μl luciferin stock using the Luminoskan RS apparatus (Labsystems). Normalisation of the experimental reporter was achieved by quenching of the firefly luciferase reaction and measurement of *Renilla* luciferase luminescence.

Oocyte nuclei were microinjected with 18 nl water containing 300–500 pg circular firefly test plasmid, plus one third this amount of control *Renilla* luciferase plasmid, with or without transcription factor mRNA. After culturing overnight in MBS, they were processed for luciferase activity as detailed above.

### Cloning and characterisation of the *Xsox17α*_*1*_ gene

The *Xsox17α*_*1*_ gene was isolated by screening a *X. laevis gilli* PAC library (RZPD) with an *Xsox17α*_*1*_ cDNA probe. One clone (BUMSP710J2012Q3) reacted strongly with this probe. A positive Not 1 fragment was subcloned into Bluescript and this sequence encompassed all 12 kb of 5′ upstream sequence present in the PAC, the transcribed region itself and 3 kb downstream of the 3′ UTR.

### Transgenic constructs and mutagenesis

Mutant promoter constructs were created by hybrid overlap extension PCR, using a series of external and overlapping internal primers. Primer sequences were as follows: B1 ext up 5′CAACACTCACATTC 3′. B1 T-box ext down 5′CTTGAGAATGGGACTGTGTTAACAAACAATGATGATCAGAACTCTGG 3′, Sox A int up 5′ CTTGGGAACTAGTTGTGGATC 3′, B1 Sox A int down 5′GATCCACAACTAGTTCCCAAG 3′, B1 Sox B int down 5′ CTTGAGAATGGGACTGTGTTAACAACCATGGATGGTGTGAACTCTGG 3′, B1 Sox B + T-box ext down 5′CTTGAGAATGGGACTGTTTTAACAACCATGGATGATCAGAACTCTGG 3′. Underlined text denotes mutated sequence. External primers had 5′ *Sac1* and 3′ *Kpn1* extension sequences. Amplification was performed over 20 cycles with 55 °C annealing temperature. Products were Qiagen column purified, cut with restriction enzyme, and cloned into pGL3basic-act-luc (transient assay) or pGL3basic-act-mgfp5 (transgenic assay).

### Electrophoretic band-shifts (EMSAs)

EMSAs were performed using VegT protein synthesised in vitro in the rabbit reticulocyte system. 10 pmol single-stranded DNA oligo was 5′ end-labelled with 20 μCi ^32^P γ-ATP using T4 polynucleotide kinase. Forward and unlabelled reverse strands were annealed by heating to 90 °C for 5 min, followed by slow cooling overnight. DNA was recovered by ethanol precipitation and resuspended in TE buffer. 15 μl binding reactions contained 1 μl in vitro translated protein, 2 μg [poly dI.dC].[poly dI.dC], 3 μl MDB buffer (20 mM HEPES, pH 7.9, 100 mM KCl, 12.5 mM MgCl_2_, 0.1 mM EDTA, 17% glycerol w/v, 2 mM DDT). The probe was added following 10-min incubation at 30 °C, followed by a further 10-min incubation. Control binding reactions included a 50-fold excess of unlabelled specific competitor probe. Samples were analysed on a 15% polyacrylamide gel at 4 °C (200 V, 2 h). Gels were dried and autoradiographed or analysed with a phosphoimager. Probe sequences used were: B1 5′-TGTCCAGAGTTCACACCATCATTGTTTGTTA-3′, T-box mutant 5′-TCTCCAGAGTACGCACATTCATTGTTTGTTA-3′, T-box consensus 5′-TCTCCAGAGTTCACACCTTCATTGTTTGTTA-3′. Underlined text denotes T-box half site and variations thereof.

### Chromatin immunoprecipitation (ChIP)

30 gastrula stage embryos were fixed in 1% formaldehyde for 10 min at room temperature, and fixation reversed by addition of 125 mM glycine for 30 min. Embryos were washed in MMR (25 mM NaCl, 0.5 mM KCl, 0.25 mM MgCl_2_, 0.25 mM CaCl_2_, 1.25 mM HEPES, pH 7.5), and homogenised in 500 μl low salt extraction buffer (25 mM Tris–Cl, pH 7.5, 70 mM KCl, 1 mM EDTA, 20% glycerol, 5 mM DTT + protease inhibitors). Shearing was performed by sonication at full power for 6 × 10 s with 2-min breaks on ice. Shearing efficiency was assessed by agarose gel electrophoresis following reversal of cross links by the addition of 200 mM NaCl and incubation at 65 °C for 5 h, followed by proteinase K treatment, phenol extraction and ethanol precipitation. This preparation also yielded input DNA for quantification of PCR reactions.

100 μl sheared chromatin preparation was diluted with 100 μl IP buffer (50 mM Tris–Cl, pH 8, 100 mM NaCl, 2 mM EDTA, 1 mM DTT, 1% NP-40 + protease inhibitors) and pre-cleared with 40 μl protein A agarose (Sigma) for 2 hrs at 4 °C. Supernatant was incubated with 4 μl serum for 2 hrs at 4 °C. A no antibody control was also included. 40 μl 50% slurry protein A agarose pre-saturated with 1 mg/ml BSA and 0.3 mg/ml salmon sperm DNA in IP buffer was added, and reactions incubated overnight at 4 °C. Beads were washed successively in IP buffer plus 0.1% sodium deoxycholate, IP buffer with 500 mM NaCl, IP buffer with 250 mM LiCl, and TE (10 mM Tris, pH 8, 200 mM NaCl). Following a pulse spin, 250 μl elution buffer (1% SDS, 0.1 M NaHCO_3_) was added to the beads and elution repeated until a 500 μl volume was obtained. Cross-links were reversed and DNA recovered as described above.

PCR was performed as described previously. 32 cycles of amplification were used. Primer sequences are as detailed below:

**Table:** 

C3B1 F	5′ GCCAATAGACACCTTTCTAG 3′
C3B1 R	5′ GAGAATGGGACTGTGTTAAC 3′
Xsox17α ORF F	5′ GGACGAGTGCCAGATGATG 3′
Xsox17α ORF R	5′ CTGGCAAGTACATGTGTCC 3′
Xom Promoter F	5′ TGTTGGCTGAGTAGGAATGAGAGG 3′
Xom Promoter R	5′ AGGCAGAGATCAGTACCACCT 3′

The Xom primers are from Messenger et al. (2005).

## Results

### Structure of the *Xsox17α*_*1*_ gene

The *Xsox17α*_*1*_ gene was isolated from a *X. laevis gilli* PAC library (RZPD) using an *Xsox17α*_*1*_ cDNA probe. A Not 1 fragment that encompassed all 12 kb of 5′ upstream sequence present in the PAC and 3 kb downstream of the 3′ UTR was subcloned into Bluescript. The transcribed sequence contains a single intron of 705 bp, starting in codon 119. The upstream region was sequenced up to − 9.5 kb, although sequence from approximately − 7.7 to − 9.2 kb was highly repetitive and proved unsequenceable. The transcriptional start site was determined by primer extension (data not shown).

In parallel, an *Xsox17β* phage λ clone with 6 kb of 5′ upstream sequence was isolated and all of its 5′ regions sequenced. Since the alpha and beta genes have identical early expression patterns, a comparison was made of the proximal 5′ promoter regions (Supplementary Fig. 1). There are many conserved features, including motifs for binding several transcription factors known to be relevant to endoderm development (Homeodomain, SMAD, T-box and Sox proteins), as well as a GA-rich region at about − 1860 bp. No other *Xsox17β* or *Xsox17*α_*2*_ clones were identified in any library.

### Transgenic analysis of the promoter of the *Xsox17α*_*1*_ gene

**Fig. 1:**
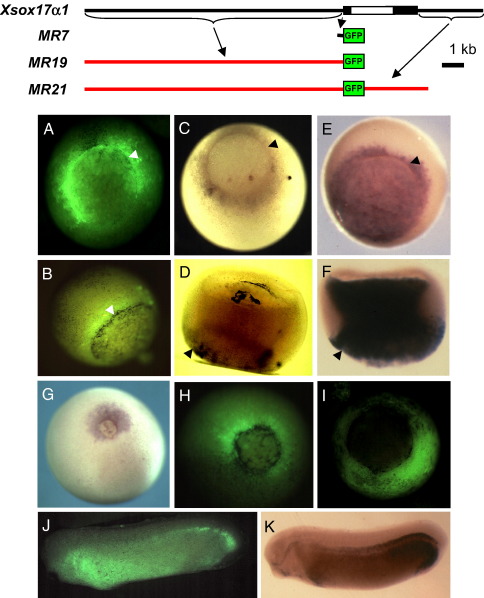
Initial transgenics made with large fragments of the *Xsox17α*_*1*_ promoter. Top: Map of the endogenous gene, with its single intron, and below are maps of two GFP constructs with large fragments of the *Xsox17α*_*1*_ promoter (red), and also a small promoter fragment with just 260 bp of upstream sequence (black). (A, B) Fluorescent expression of the MR21 construct in stage 10.5 gastrulae. (C) In situ hybridisation to GFP mRNA from MR19 in an embryo similar to that in panels A, and (D) an optical section of this embryo after clearing. (E, F) In situ to the endogenous *Xsox17* transcripts for comparison. The blastopore lip is marked by an arrowhead in panels A–F. At stage 12, the endogenous gene is expressed as in panel G, and the MR21 GFP is shown in panel H. A minimal promoter, with only 260 bp of 5′ sequence is not expressed in the vegetal region (I), but it is expressed elsewhere. (J) MR21 is expressed throughout the endoderm at tailbud stages, just like the endogenous gene, as shown in an in situ hybridisation (K). The arrowheads mark the blastopore.

Initially the entire 12 kb upstream of the *Xsox17α*_*1*_ gene was fused to a GFP expression cassette to give MR19. A second construct was made which also contained 3 kb downstream of the transcribed sequence. This was placed downstream of the GFP cassette (MR21) (Fig. 1, top panel). When the method of Kroll and Amaya (1996) was used to make transgenics containing these sequences, GFP expression was observed throughout the pre-endoderm of the gastrula, including the superficial region surrounding the blastopore lip, which is known as the involuting or extra-blastoporal pre-endoderm (Figs. 1A, B). Expression was screened by GFP fluorescence, which under-reports in the vegetal region (Ahmed et al., 2004), and it was confirmed by in situ hybridisation to GFP mRNA (C) which also under-reports in vegetal tissues, but to a much lesser degree if suitable protocols are used (Sasai et al., 1996). Bearing this in mind, Fig. 1 shows that the pattern at early and late gastrula was similar to the in situ hybridisation pattern of expression of the endogenous gene (E, F). Comparison of MR19 and MR21 transgenics indicated that they were similar in the gastrula (H), but that the 3′ region of MR21 reduced expression outside the endoderm at later stages. This sequence has not been investigated further. The 260 bp minimal promoter was negative in terms of endodermal expression (I), but was expressed elsewhere. Typical numerical results for the transgenics are shown in Supplementary Tables 1 and 2. Constructs either consistently gave endodermal expression in the gastrula, or consistently failed to do so.

There was later expression of GFP in the pharynx and hindgut of the tailbud tadpole (Supplementary Fig. 2). This corresponds to regions where the endogenous gene is expressed, as judged by RT–PCR (Clements et al., 2003), but there was no expression in the region of the developing gall bladder, where the gene is strongly expressed at this stage (Hudson et al., 1997; Zorn and Mason, 2001). Later there was robust expression in the foregut and proctodeum, as well as in the pancreas (Supplementary Fig. 2). Zorn and Mason (2001) did not report expression in the pancreas at these stages, so this may represent either incorrect regulation or persistent, stable GFP expression. There was also expression in lateral line organs and in the brain (fore/mid brain boundary, cranial nerves). It is not known if *Xsox17α*_*1*_ is expressed in all these regions. However there was GFP expression in the lens, and RT–PCR shows that *Xsox17* is expressed there, as are other Sox genes (data not shown) (Schlosser and Ahrens, 2004; Zygar et al., 1998). There was also expression in the olfactory organs, which has been shown for the endogenous genes by in situ hybridisation (Zorn, personal communication), as well as in skeletal elements of the developing limbs.

We have focused on blastula to early gastrula stage expression of the promoter because this is the time that the endodermal domain, marked by *Xsox17* expression, is mapped out. To define the regions responsible, we first showed that there were no sequences promoting endodermal expression in the single intron or the 3 kb downstream of the cDNA sequence (not shown). We then showed that although the 260 bp proximal region contains sequences conserved in the *Xsox17β* promoter, including T-box sites (Supplementary Fig. 1), it drove expression everywhere except the future endoderm. It is thus expressed only where *Xsox17α*_*1*_ is not. However, later it gave expression in the pharynx, duodenum, pancreas, stomach and proctodeum, as illustrated for longer promoters in Supplementary Fig. 2. The 6 kb upstream of *Xsox17β* also did not drive gastrula endodermal expression (not shown) and we could not identify any *Xsox17*α_*2*_ clone. Thus a comparison of endodermal elements in the three *Xsox17* genes could not be made. While it is possible to compare sequences with those in *Xenopus tropicalis*, any interpretation would be dubious since the detailed regulation of *X. tropicalis* has not been studied in this species.

**Fig. 2:**
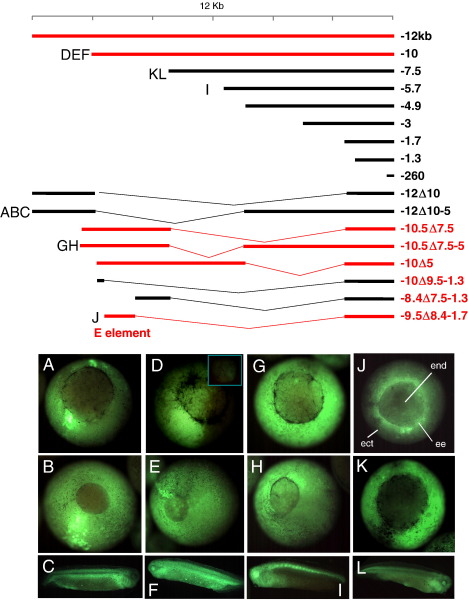
Transgenic expression of deletion constructs. Top: Diagrams of a series of 5′ and internal deletion fragments fused to GFP, as in Fig. 1. The endodermal element is marked and fragments giving vegetal expression in the early gastrula are colored red. Below is GFP expression in various transgenics, with the corresponding expression panels indicated on the left of the constructs. (A, D, G, J, K) are stage 10.5; (B, E, H) are stage 12; (C, F, I, L) are tailbud stages. (A–C) Construct − 12Δ10-5 is negative in early endoderm. (D–F) Positive construct − 10; the embryo in (D) is a hemi-transgenic, providing a good control for the background fluorescence; the insert panel is the animal pole at the same exposure. (G–H) Positive construct − 10.5Δ7.7-5. (I) Construct − 5.7 is positive in the later foregut, as well as in the axis, like other 5′ constructs; (J) − 9.5Δ8.4-1.7 is positive in the involuting and non-involuting endoderm, whereas − 7.5 is not (K), but it gives low-level expression in the posterior endoderm of the tailbud (L). In panel J, the main tissues of the early gastrula are marked: end, endoderm; ect, ectoderm; ee, the extra-blastoporal endoderm, which involutes over the blastopore.

A wider screen for regulatory elements in *Xsox17α*_*1*_ was made with a deletion series (Fig. 2). Early endodermal expression required regions about 9 kb from the start of transcription. This defined a 1.1 kb “Endodermal element”, which alone drove strong GFP expression in the early vegetal/endoderm region. Internal deletions show that the E-element can drive strong endodermal expression when placed on the first 1.7 kb 5′ to the start of transcription, which itself only gave low GFP expression in non-endodermal regions of gastrulae and later stages (Fig. 2J). Later experiments, described below, investigated sub-fragments of this region fused to a basal promoter from a *Xenopus* cytoskeletal actin gene that is expressed in embryonic striated muscle (Ahmed et al., 2004; Latinkic et al., 2002), showing that the E-element does not require sequences in the proximal region of the promoter for endoderm-specific expression. Thus, in the context of the sequences studied, the E-element is the only region both necessary and sufficient to direct expression in the progenitor of the endoderm and in the early gastrula.

### Dissection of the E-element

**Fig. 3:**
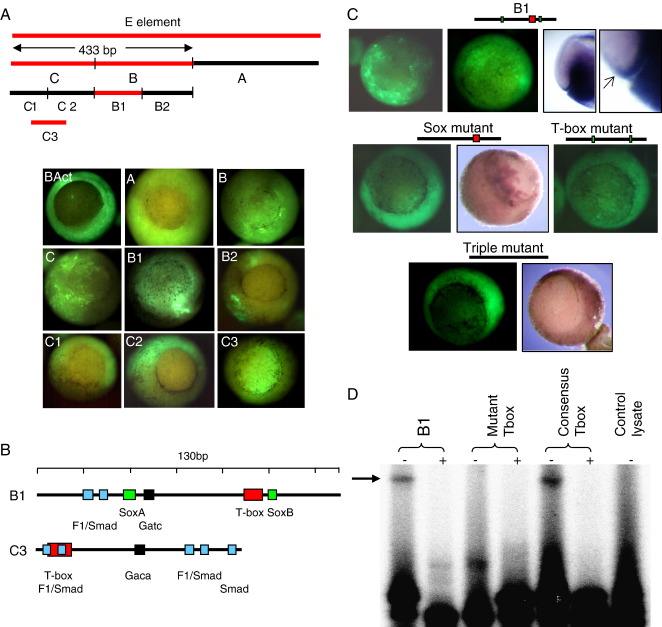
(A) Dissection of the E-element. At the top are the sub-regions of the E-element that were tested in GFP transgenics, attached to a cytoskeletal-type muscle actin basal promoter. All constructs except C3 overlapped to avoid disrupting a possible control element. Images of the transgenics are shown below, labelled by construct, the basal promoter alone being labelled BAct (Latinkic et al., 2002). Expression of the entire E-element is shown in Fig. 2J. (B, C) Mutational analysis of the B1 element. In panel B are maps of selected transcription factor binding sites in B1, and also C3. The sequences of B1 and C3 are shown in Supplementary Fig. 3. In panel C is a transgenic analysis of mutants of B1 in which the transcription factor binding sites were disrupted. The green panels show GFP fluorescence and the others show in situ hybridisation to *GFP* mRNA. The third B1 panel shows a cleared embryo, with GFP expression in the deep involuting endoderm and also the extra-blastoporal, epithelial, involuting endoderm, enlarged in the fourth panel. The arrow indicates the blastopore lip. (D) Electrophoretic mobility shift analysis of B1 T-box motif. EMSA assays were conducted with the variant VegT-binding sequence in B1, with a mutant of it and with the consensus sequence in the Derrière gene. In each case, the reactions were performed with or without competitor (±).

The E-element was divided into three sub-fragments (A–C), which were coupled to the basal cytoskeletal actin promoter and tested in transgenics (Fig. 3A). Only B and C gave vegetal expression. Each was further divided into two approximately equal fragments. For B, only B1 drove vegetal/endodermal expression, but neither C sub-fragment did. However a sub-fragment from the central region of C gave vegetal expression, albeit with relatively high animal expression (Fig. 3A). Since the C1 and C2 fragments were made with an overlapping 18 bp region to avoid disrupting a regulatory sequence, there clearly are sequences within C1 and C2 which must co-operate to give the endodermal expression of C3.

Thus two independent *Xsox17α*_*1*_ control regions, B1 and C3, are capable of driving expression in the presumptive endoderm of the gastrula. The expression of B1 (Figs. 3A, C) is notably similar to the endogenous *Xsox17* genes (Figs. 1E, F). The GFP expression in the vegetal regions is detectable by early stage 10, the onset of gastrulation. Given that GFP requires an hour or more to mature and fluoresce, vegetal expression of the transgene must begin in the blastula, during the initiation and/or establishment phases of *Xsox17* regulation (Clements et al., 1999; Yasuo and Lemaire, 1999). As discussed earlier, GFP under-reports in the vegetal pole (Ahmed et al., 2004), so any detectable vegetal fluorescence indicates strong actual expression.

### Transgenic analysis of the T-box and Sox sites in element B1

B1 contains several consensus binding sites for known endodermal regulators (Fig. 3B; Supplementary Fig. 3), including a variant T-box binding site (core CACCA rather than CACCT) and two canonical Sox sites (core CATTG). To test if the variant T-box binds VegT, we performed electrophoretic gel mobility shift assays (EMSA), comparing the binding of VegT to this sequence and the consensus sequence identified in the *Derrière* promoter (White et al., 2002). Fig. 3D shows that the binding of the two is similar. As a control, no binding was observed to a sequence containing a mutant site (core CACAT).

The T-box and the two Sox sites were mutated and their efficiency in transgenics assessed (Fig. 3C). The numbers of transgenic embryos in a typical experiment are shown in Supplementary Table 2. The double Sox site mutant gave vegetal pole expression which was somewhat less intense than that produced by the wild-type B1. There was also raised relative expression in the animal pole, suggesting that these sites may play a possible repressive role in the non-endodermal region. The T-box mutant also gave vegetal expression, although again it appeared to be at level lower than from an unmutated B1 element, although this is difficult to quantify. When the T-box site was mutated in conjunction with the Sox sites (Fig. 3C, triple mutant), expression of GFP was excluded from the endodermal domain.

### Transient transgenic analysis of B1 and C3

Although in transgenics the constructs either gave endodermal expression, or they did not, the results are not quantitative and the variability in the absolute level of expression from embryo to embryo makes it difficult to compare constructs objectively. Transient transgenesis using the quantifiable reporter luciferase circumvents this problem. DNA may be introduced into different parts of the embryo, or injected it into a region where *Xsox17* is not expressed (i.e. the animal hemisphere) with or without mRNAs encoding specific regulatory molecules, like VegT. To enable this, promoter elements, together with the cytoskeletal actin basal promoter, were cloned into pGL3basic (Promega), which lacks eukaryotic promoter and enhancer sequences. The DNA was linearised for injection because sometimes this is necessary for expression (Wilson et al., 1986).

**Fig. 4:**
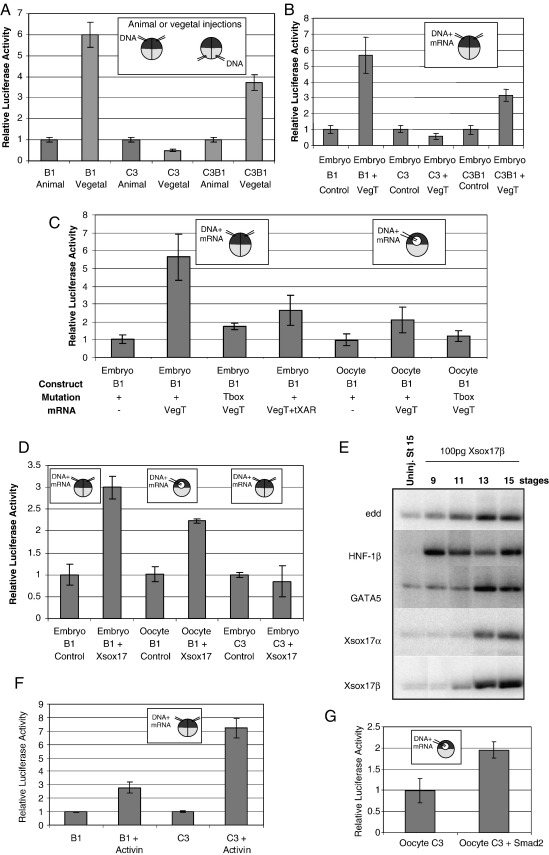
Transient transgenic analysis of the B1 and C3 promoter elements using luciferase constructs. In all experiments, the vegetal samples injected with DNA constructs or the animal poles injected with DNA plus mRNA are normalised relative to control animal hemisphere expression of the same DNA construct alone (set to 1.0). Each individual test plasmid firefly luciferase measurement is first normalised with respect to an internal *Renilla* luciferase control. (A) Animal versus vegetal expression of the endodermal elements. (B) Response of endodermal elements to VegT in animal hemispheres. (C) Effect of mutating the T-box site on B1 response to VegT. The first four tracks show expression in the embryonic animal hemisphere, the last three show expression in the oocyte to measure direct effects of VegT (see text). Co-injection into the embryo of mRNAs encoding VegT and dominant negative Actin receptor (tXAR) demonstrates reduced induction by VegT when TGF-β signalling is blocked. (D) Responses of B1 and C3 to *Xsox17* mRNA. (E) RT–PCR showing time course of gene induction by *Xsox17* mRNA in animal caps. Expression in control animal caps is the same at all stages. (F) Activin responsiveness of B1 and C3 in animal caps. The constructs, with and without 20 pg Activin mRNA were injected into animal poles and the embryos analysed at stage 10.5. (G) C3 was injected into the oocyte nucleus together with 200 pg activated *Smad2* mRNA.

First constructs containing B1 and C3 elements were injected into vegetal and animal hemispheres, to establish whether the transient expression mirrors the natural high expression of *Xsox17* in the vegetal compared to the animal pole. Fig. 4A shows that B1 is much more active in the vegetal than the animal hemisphere, in agreement with the transgenics. This is also true, to a reduced degree, of the C3B1 combined fragment. Surprisingly, C3 showed reduced expression in the vegetal relative to the animal hemispheres. This may partly be because these results are expressed as a ratio of animal to vegetal expression. High expression in the animal hemisphere was also seen in GFP transgenics where we simply scored for vegetal expression. Presumably inhibitory elements are lacking.

B1 contains T-box and Sox-binding sites that were essential for high endodermal expression in transgenics. We tested the functionality of the T-box site by co-injecting B1 DNA plus VegT mRNA into the animal pole, where VegT mRNA is normally present only at a low level. The basal promoter did not respond to VegT in these assays (not shown). VegT stimulates B1 activity by an amount comparable to that produced by vegetal compared to animal hemisphere injections (Fig. 4B). In contrast, C3 is inhibited slightly, even though it contains a consensus T-box site core sequence (CACCT). Flanking nucleotides of this site are divergent (AGACACCT; consensus TCACACCT), and White et al. found that this sequence bound VegT only weakly in EMSAs (White et al., 2002). The C3B1 fragment produced an intermediate result. Thus B1, but not C3, responds positively to VegT and the responses of B1 and C3 to VegT mirror their activity in vegetal versus animal poles.

Since VegT, directly or indirectly, induces expression of many transcription factors which are part of the mesendodermal gene network, including other T-box proteins (Eomes and Xbra), we sought to confirm that VegT directly interacts with B1 in two ways. It is of course known that the endogenous *Xsox17* genes are direct VegT targets from experiments with inducible VegT fusions (Clements et al., 1999; Clements and Woodland, 2003; Yasuo and Lemaire, 1999). Firstly we mutated the T-box site, which greatly reduced, but did not eliminate the stimulation by VegT (Fig. 4C, lane 3). This implicates a T-box protein in the regulation. Secondly, we examined expression in oocytes in response to co-injected VegT (White et al., 2002). The amount of transcription seen when DNA is injected into an oocyte nucleus is comparable to an embryo, this being supported by stores of chromosomal and transcriptional proteins. However there is only one nucleus in an oocyte, so the amount of downstream nuclear gene expression that the oocyte can support over the time course of our experiments is negligible, even over an 18-h time course (White et al., 2002). One hour of transcription in the late blastula would be equivalent to 10,000 h for the oocyte, equating to well over a year. This suggests that any effects of VegT on the co-injected DNA are directly dependent on VegT itself. Fig. 4C (last three lanes) shows that VegT stimulates B1 transcription in oocytes and that this is absent for the T-box mutant. The stimulation is less in oocytes than in embryos, presumably because cooperating genes are not significantly induced by VegT in oocytes. For example endogenous *Xsox17* expression is dependent both on VegT itself and on the TGF-β signalling that VegT induces (Clements et al., 1999; Clements and Woodland, 2003; Xanthos et al., 2001). To test if the B1 element responds to TGF-βs downstream of VegT (directly or indirectly), we blocked TGF-β family signalling by co-expressing VegT and a truncated Activin receptor (tXAR) in animal hemispheres (Hemmati-Brivanlou and Melton, 1992). Fig. 4C, lane 4, shows that the stimulation of B1 by VegT is reduced, but not eliminated. These results are consistent with the proposal that B1 responds to VegT directly via its T-box site, but that co-operating molecules downstream of VegT-induced TGF-β signalling are also necessary for the full VegT induction in an embryo. Consistent with this interpretation, in oocytes there is no VegT stimulation of the B1 element with a mutated T-box because VegT cannot induce downstream genes such as TGF-β signals (Fig. 4C, last lane).

Transgenesis also showed that B1 contained functional Sox-binding sites and it was already known that the endogenous *Xsox17*α genes are direct Xsox17 targets (Sinner et al., 2004). Fig. 4D shows that in the animal hemisphere B1 responds strongly to Xsox17 and expression in the oocyte supports the view that this is a direct effect. Xsox17α and β induce themselves and each other (Sinner et al., 2004), but the autoinduction is quite slow, as shown in Fig. 4E. The luciferase inductions fitted this because there was no significant induction by Xsox17 at stage 10.5 (not shown), but it was clear by stage 17 (8 h later at 23 °C). Xsox17 probably normally co-operates with other factors that are maternal or downstream of VegT on certain promoters. However, this only applies to a subset of promoters, whereas other genes like *Hnf-1β* do not show this delay, presumably because any co-operating factors are already present in the animal hemisphere. It is likely that when *Xsox17* alone is expressed ectopically, it slowly induces the co-operating factors needed for auto-induction, eventually establishing a partial endodermal gene network. Normally the cooperating molecules are maternal or are induced by VegT. In contrast to B1, C3 did not respond to Xsox17, as expected from its lack of consensus Sox sites.

While C3 was activated by neither VegT nor Xsox17, it contains several consensus Smad-binding sites coupled to those for co-operating FoxH1 (Fast1), suggesting that it might respond to TGF-βs like Activin. Fig. 4F shows that this is indeed the case, suggesting that it might mediate the TGF-β response of Xsox17α_1_. In oocytes, C3 is induced by constituently active Smad2, indicating that the induction by Activin is direct (Fig. 4G). In contrast, B1 shows very little response to Activin in embryos. This is surprising since many gene products that induce Xsox17α_1_ are downstream of Activin, including the *Xsox17s* themselves. While in this experiment the luciferase was assayed before Xsox17 would have exerted its slow effect in animal caps (see above), there is little effect even at later time points (not shown), which suggests that the level of Xsox17 induced by Activin is too low to produce a strong induction without co-operating VegT. We show below that VegT and Xsox17 co-operate in regulating B1, and synergy between VegT and Activin was previously noted (Clements and Woodland, 2003).

### Mutational analysis of B1 shows that one Sox site is most important and it co-operates with the VegT site

**Fig. 5:**
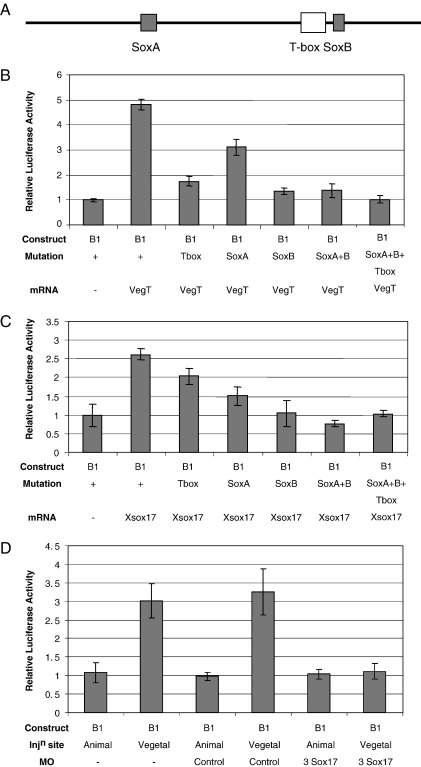
Mutational and anti-sense analysis of B1. (A) Position of the T-box and Sox response elements in B1. (B) Responses of B1 mutants to *VegT* mRNA, assayed at stage 10.5. All DNAs, together with the appropriate mRNAs, were injected into animal hemispheres and the luciferase activity normalised to each DNA injected alone (set to 1.0). Only the unmutated B1 without *VegT* bar is shown, since all controls were set to 1.0. (C) Response of B1 and its mutants to Xsox17α_1_, assayed at stage 17. (D) The stimulation of B1 in the vegetal compared to the animal pole is eliminated by injecting the anti-sense morpholino oligos against α_1_, α_2_ and β *Xsox17* mRNAs.

To assess the roles of the two Sox sites in B1, the response of a series of single, double and triple mutants of Sox and T-box sites was assessed by injection into animal hemispheres. Most of the response to VegT was dependent on the T-box site and the neighbouring B Sox site, whereas removal of the more distant A Sox site had much less effect (Fig. 5B). When all three sites were removed, there was no induction by VegT, and no expression in the vegetal pole. Since removal individually of either the B Sox or the T-box sites, but not the A Sox site, removed much of the response of B1, maximal induction clearly requires the presence of both B Sox and T-box sites. This suggests that there is synergy between the VegT and Xsox17 bound to these sites. The role of the two Sox sites in responses to Sox17 is examined in Fig. 5C. This confirms that Sox site B has the main activity and the T-box site is unimportant in induction by Sox17 alone.

It is possible that in the endoderm itself, the B1 Sox sites respond to Sox proteins other than Xsox17. To test this B1 DNA was injected into the vegetal pole, followed by injection of the three Xsox17 antisense morpholino oligos (MOs) that block translation of the three *X. laevis Xsox17* mRNAs (Clements et al., 2003). The enhanced activity of B1 in vegetal poles, as compared to animal, was eliminated, whereas a control MO had no effect (Fig. 5D). This confirms that the endogenous Xsox17s are responsible for the high activity of the B1 element in the developing endoderm.

### The B1 element is bound to Xsox17 in vivo

**Fig. 6:**
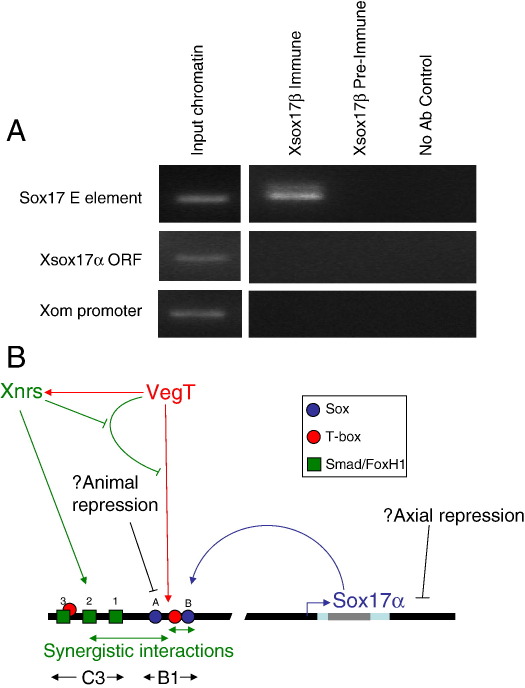
(A) Chromatin immunoprecipitation of the B1 element. Chromatin from stage 11 gastrulae was precipitated with an anti-Xsox17β antiserum, the DNA extracted and subjected to PCR using primers to the B1 plus C3 elements, the *Xsox17α* ORF and the *Xom* promoter, which should not be regulated by Xsox17. (B) Summary of the regulation of the *Xsox17α*_*1*_ promoter. VegT induces *Xsox17α* directly, via the T-box half site in B1, as well as nodal-related proteins (red). However, the Xsox17 induction becomes inhibited by a VegT-derived inhibitor. The inhibition is over-ridden by TGFβ signals, induced by VegT. This may simply be by the positive induction of C3 via FoxH1/Smad sites (green). Xsox17 autoregulates itself synergistically through Sox binding sites (blue) and the adjacent T-box half site (red). In addition, these Sox sites might respond to repressive members of the Sox family, restricting expression to the vegetal pole.

To confirm that the Sox sites in B1 are functional we needed to show that they are occupied by Xsox17 in the living embryo. We therefore performed a chromatin immunoprecipitation (ChIP) on mid-gastrulae stage embryos. Chromatin was isolated, sheared to DNA fragments of about 500 bp and immunoprecipitated with an *Xsox17β* anti-serum (Clements et al., 2003). Fig. 6A shows PCRs from several regions in the resulting genomic DNA. The Xsox17 B1 plus C3 element was clearly precipitated by the anti-serum, but not pre-immune serum. Conversely, the *Xsox17α*_*1*_ ORF was not precipitated, as expected since it is about 9 kb distant from the E-element. A second control was to probe for an enhancer previously used for immunoprecipitation with an Xbra anti-serum (Messenger et al., 2005). This ventrally expressed gene should not be regulated by Xsox17 and indeed the promoter element fails to precipitate with *Xsox17β* antiserum. Therefore, as expected from the transgenic data, the E-element is bound to *Xsox17β* in vivo.

## Discussion

Expression of the *Xenopus Xsox17* genes defines the endodermal territory in the mid-blastula, when cells start rapid transcription and make the initial decisions to become endoderm. For this reason, we have focused on the regulation of one of these genes, *Xsox17α*_*1*_, in the blastula and early gastrula. We have been able to identify two regulatory elements at approximately minus 9 kb that drive transcription in the future endoderm at blastula and gastrula stages. Study of the endogenous genes has suggested that the regulation of the *Xsox17* genes is very dynamic, passing rapidly through initiation, establishment and maintenance phases (see Introduction). In the initiation phase, *Xsox17α*_*1*_ is a direct target of the localised maternal transcription factor VegT, in the establishment phase it becomes signal-dependent and eventually maintenance is cell-autonomous. One would hope to find the basis of these regulatory processes in the *Xsox17α*_*1*_ promoter.

### Activity of the C3 endodermal element

Of the two adjacent elements capable of driving endodermal expression, B1 responds strongly to VegT and Xsox17, and C3 to Activin, but not to VegT. We have principally focused on B1, but we have made a preliminary analysis of C3. This 89-bp region must contain cooperating elements since two fragments, each containing half of C3 plus an 18-bp overlapping region, were negative in terms of endodermal expression. The Activin responsiveness of C3 correlates with its possession of three separate pairs of closely associated FoxH1/Fast1 and Smad sites, which are known direct effectors of Activin/Nodal signalling (Chen et al., 1997; Germain et al., 2000). However, Activin induces the expression of many downstream molecules, which could then be responsible for indirect induction of this element. To prove the effect was direct we showed that C3 is stimulated by activated Smad2 in the oocyte. This indicates a direct effect, since the single nucleus of this cell has 10,000-fold less transcriptional capacity to induce downstream effectors of Activin/Smad action than does the early gastrula.

Maternal depletion of FoxH1/Fast1 by Kofron et al. (2004) showed that it was not essential for *Xsox17*α expression. However, as they point out, *Xenopus* embryos also express XFast-3, which binds to the same target sequence (Howell et al., 2002), and could therefore redundantly regulate *Xsox17* through the same promoter elements.

C3 responds strongly to Activin, but with respect to vegetal expression and response to VegT, its behaviour is paradoxical. It is surprising that it does not respond to VegT in the embryo, both because C3 contains a consensus T-box core sequence and because VegT induces the expression of TGF-βs that, like Activin, act through Smads 2/3; indeed VegT depends on this signalling for its overall biological effect. The T-box site in C3 entirely overlaps a Fast1/Smad pair of sites, which may have a bearing on the fact that VegT actually inhibits C3 basal expression, just as its expression is inhibited in the vegetal pole compared to the animal. This is consistent with the observation that removal of the T-box site removes the inhibition (data not shown). C3 was identified by its ability to direct expression in the vegetal pole of transgenics, however here we simply scored expression in the vegetal pole itself, disregarding animal regions. Conversely, the luciferase measurements are of the ratio either of vegetal to animal expression or of VegT stimulated to control expression in the animal region. Therefore a high level of animal expression would mask vegetal activity of the promoter. However, it is important to note that both the VegT induction and vegetal expression are consistent. We believe that the response to TGF-βs is the important property of C3 and other problems are introduced by looking at small regulatory regions in isolation, where synergising and inhibitory effects are absent.

Previous work has shown that *Xsox17* expression switches from direct VegT response to TGF-β signal dependence. C3 is a therefore a good candidate for controlling the signal-dependent process. It is conceivable that the inhibitory effects we notice bear on the hypothetical inhibitor that make *Xsox17* signal dependent in the establishment phase of endoderm development.

### The activity of the B1 endodermal element

The activity of B1 is more straightforward. It is highly expressed in the presumptive endoderm in transgenics and it is much more highly expressed in the vegetal than the animal hemisphere in transient assays. It is also strongly stimulated by VegT. It contains a divergent T-box site which binds VegT in vitro and which mutation shows is partially responsible for the VegT stimulation in the embryo. Expression in oocytes shows that B1 responds directly to VegT through this site, although the stimulation is less than in embryos. This correlates with the fact that blocking TGF-β signalling in embryos with a truncated Activin receptor reduces VegT stimulation to about the oocyte level. Thus B1 responds directly to VegT and synergistically to other molecules that are downstream of the VegT-induced TGF-βs, which would principally be Nodal-related signals. These synergistic molecules are the Xsox17 proteins themselves in an autoregulatory loop.

B1 responds directly to Xsox17. Other molecules, such as Gata 4–6, are downstream of Xsox17 and could in principle regulate B1. Although B1 contains a possible variant Gata-binding site, mutation of the two consensus Sox sites removes much of the activity of B1, and the triple Sox/VegT mutant is unresponsive both to VegT and vegetal pole expression. Further, mutation of the possible Gata site has no effect on the response of B1 to VegT (not shown). The direct action of Xsox17 on B1 was confirmed by transcriptional assays in oocytes. These observations fit with the fact that Xsox17 auto-induces itself (Sinner et al., 2004), but endogenous *Xsox17* genes are very slow to be induced when *Xsox17* is expressed ectopically in the animal cap, and this is equally true of the B1 regulatory region. Previously we have found the same for a direct Sox inductive site in the *Endodermin* promoter (Ahmed et al., 2004). In fact, we believe that the Sox sites in B1 are already needed at blastula stages because they are required for full vegetal expression of the GFP transgene as early as the onset to gastrulation, judged by GFP fluorescence. Since GFP takes several hours to mature (Davis et al., 1995), there must have been an earlier requirement for the Sox factors. We have argued previously, in connection with the *Endodermin* promoter, that Xsox17 must normally co-operate with other molecules found in the vegetal pole. In the animal cap Xsox17 presumably establishes the endodermal gene network more slowly than its normal establishment by multiple maternal inputs (Sinner et al., 2006). Since B1 is rapidly induced by VegT, and this depends largely on the Sox sites and *Xsox17* expression (as shown by Sox site mutants and blocking Xsox17 action with combined Xsox17 group morpholinos), it seems that VegT is able to co-operate with Xsox17 to produce more rapid Sox action.

The kinetics of reporter expression suggest that the VegT and the T-box site are not simply needed at the very onset of endodermal gene expression in the initiation phase, but that this continues into gastrulation during the establishment phase. It is provocative that the Sox site of principal importance in B1 is very near to the VegT site, suggesting a direct interaction between VegT and Xsox17, although we have been unable to detect this by immunological co-precipitation.

### The role of *Xsox17* auto-induction in the endodermal network

The idea that a key endodermal gene, *Xsox17*, induces itself is attractive because it would give stability to *Xsox17* expression and hence to the endodermal network. The requirement for signalling also adds a fail-safe for cells that become misplaced from the endodermal domain, but still initiate direct VegT-dependent endodermal gene expression. These conform to the fate of their neighbours (Clements et al., 1999; Clements and Woodland, 2003; Wardle and Smith, 2004; Wylie et al., 1987; Yasuo and Lemaire, 1999). However, blocking Xsox17 action with morpholino oligos has no effect on *Xsox17* expression, even though other Xsox17 targets, including *HNF1-β*, *Endodermin* and *Gata5*, are down-regulated (Clements et al., 2003; Sinner et al., 2006). The most obvious reason for this paradox is redundancy. For example *Xsox17* can be induced by Gata4, 5 and 6 and the first two are only partially and the latter not at all affected by Xsox17 morpholinos (Clements et al., 2003, and unpublished data). *Xsox17* is a direct Gata6 target (Afouda et al., 2005), but while B1 and C3 elements contain possible divergent Gata sites, mutating these in B1 does not prevent its response to Gata4–6. Furthermore, only the Sox and T-box sites of B1 are essential for expression in the endoderm. While other *Xsox17*-inducing factors, like Mix/Bix proteins, could be relevant, another candidate is maternal Xsox7, which is localised in the vegetal pole (Zhang et al., 2005). Indeed, we have shown that overexpression of Xsox7 in the animal hemisphere results in the induction of Xsox17, although again this is slow, as with Xsox17 itself (data not shown).

Nevertheless, the case for *Xsox17* auto-regulation is compelling, now being based on three independent lines of evidence. Firstly, Xsox17 induces itself in ectopic expression experiments, and this is direct (Sinner et al., 2004); secondly, there are essential Sox sites in a promoter element that drives endodermal gene expression in transient and true transgenics; thirdly ChIP analysis shows that the endodermal element is bound to Xsox17 *in vivo*. Based on the activity of a glucocorticoid derivative of VegT in the absence of protein synthesis, *Xsox17* is a direct target of VegT (Clements and Woodland, 2003), and our results show that it co-operates with Xsox17 (and possibly other transcription factors) to establish and amplify *Xsox17* gene expression. The fact that *Xsox17* expression is ultimately dependent on TGF-β signalling makes this amplification and stabilisation of its own expression signal-sensitive and hence subject to a community effect (Gurdon et al., 1993a,b). We propose that this involves the C3 element. A simplified version of this network is shown in Fig. 6. An important point to note is that other members of the endodermal network, like the *Mix/Bix* genes, are similar to *Xsox17* with respect to VegT induction and signal dependence.

There are still many issues to be resolved relating to the regulatory elements. A VegT-induced inhibitor of its own action in inducing *Xsox17* was postulated to explain the switch to signal dependence of VegT action, and this is still unknown. It may relate to the repressive effect of VegT and vegetal position on C3, a repression that is lifted by TGF-β signalling. There also appear to be inhibitors of expression in other parts of the embryo. For example, the loss of the Sox sites appears to increase the background expression in the animal pole. This could involve the action of the inhibitor Xsox3 (Zhang et al., 2003, 2004). At later stages. there is also expression of most transgenes in regions like the axis, where *Xsox17* is not expressed. This was not seen in a construct including 3 kb of 3′ sequence, suggesting that late inhibitory sites are present in this region.

The regulatory interactions described here are elements within the core endodermal network (Sinner et al., 2006). The form of the regulation, where VegT induces several factors, such as TGF-βs and Xsox17, and co-operates with them to regulate an important control gene, is an important recurring regulatory motif, the feed-forward motif (Mangan and Alon, 2003). In this case, it also brings about autoregulation. Autoregulatory effects of proteins in transcription factor networks are thought to have special importance. Mochizuki (2005) found that the number of possible stable states of gene networks depended only on the number of autoregulated components. Since it is reasonable to equate stable states with differentiated states, the autoregulation of a key endodermal transcription factor would be most significant in endodermal differentiation.

## Appendix A Supplementary data

Supplementary Fig. 1 Comparison of the proximal 5′ upstream regions of the *Xsox17α*_*1*_ and *β* genes. Important transcription factor consensus binding sites are indicated. The last residue, marked in blue, is the first of the transcript, determined by primer extension.

Supplementary Fig. 2 Later expression of *Xsox17α*_*1*_ transgenes. Constructs were MR19 (A–C) and N-10 (D–H): (A) stage 37; (B–C) stage 47; (D–G) stage 54; (H) stage 56. (E–F) Regions dissected out for photography: (E) stomach/duodenum/pancreas, (F) lens. (G–H) Hind legs. Note the strong yellow fluorescence of the yolky gut. Abbreviations: hl, hind legs; l, lens; ph, pharynx; pc, pancreas; pr, proctodeum; ll, lateral line; mh, mid-hind brain boundary; olf, olfactory organ; m, mouth.

Supplementary Fig. 3 Sequences of endodermal sub-elements B1 and C3. Possible Smad sites (italics), FoxH1 (synonym Fast1) sites (blue), Sox binding sites (green) and T-box half site (bold underline).

Supplementary Table 1 Typical transgenic experiment analyzing deletion mutants.

Supplementary Table 2 Analysis of normal transgenic mid-gastrula (stage 10.5) embryos and transgene expression in experiments analyzing the effects of endodermal element mutations.
