# Supplementary material for: Regulation of the Xenopus Xsox17α1 promoter by co-operating VegT and Sox17 sites
Source: Dev Biol. 2007 Oct 15;310(2):402–15. doi: 10.1016/j.ydbio.2007.07.028 (PMC2098691; doi:10.1016/j.ydbio.2007.07.028)
Supplement: Supplementary Fig. 1 — Comparison of the proximal 5′ upstream regions of the Xsox17α1 and β genes. Important transcription factor consensus binding sites are indicated. The last residue, marked in blue, is the first of the transcript, determined by primer extension. [file mmc1.doc]

**Supplementary Fig. 1**

**Comparison of proximal regions of Xsox17α and Xsox17β promoters**

α 1 .........GGGTCGTTAAAAAAGAANATAGNAGATANGGATTG...TNT 38

| | ||| | : |||:| :| |||| |:

β 1800 CATCTGGTAGCTATGGGGGAAATTACCTTAGTAACCTCGCATTGGTTTGA 1849

. . . . .

39 AAAAAAGAATATNTATGATGTAGGAGAGCTTAGGTAGTAGTAGTAGTAGT 88

| || ||| | : | ||||||| | | | ||| | | |||

1850 ATAAGAGACTGGAATATGAGCAGGAGAGAGTCTG.AATAGAAAGATGAGT 1898

. . . . .

89 AGTATAGTGGGGATATGGGTACGAGAGAGTAAAAGGA.ATTAAGTAGTAG 137

|| | | | || || || | | | | |

1899 CATAAAAAGTAGCAATAACTATACATTTTTAGCCTTACAGAGAATTATGT 1948

. . . . .

138 CTGTGAATAGGGTGCGTTAGAAGGAATGGTGAGGAGGGGCACGGGACATA 187

| | ||| |||| | | | | | || || | | |

1949 TTTTAAATGGGGTCAGCGACCCCCATTTGAAAGCTGGAAAATGTCTAGAA 1998

. . . . .

188 GAAACAGAGAGAGCCGGCGTCTAGGAACAG.......CAGAAGAGAGCGA 230

||| || | | | | | || || || ||| | |

1999 GAAGAAGGAAAATCATTCAAAAACGATAAGAAATTAATAGTGAAGACCAA 2048

. . . . .

231 GTGAAGTAAAGAGTAATGAGAACGAGACCAAAACCCTGTCCTCAAAACAC 280

|||| | || | | | ||| | | | | ||

2049 TTGAAAAGTTGCTTAGAATTGTCCATTTTATAACATACTATTAACAGCAG 2098

. . . . .

281 CTCATAGTAAATA**TAAT**GCCGAGCAAGAAAAA**GAAACATTG**CAC**GTCC**CA 330

| ||| | | | || | | |||| | |

2099 GAGGAAA**TAAT**GCATTTATCTA..AACATTGCTG**ACAATTGTT**G**GCCT**GG 2146

. . . . .

331 CGCAGTATAAGGCATACTGTATATAATAATAATTGATTATACAGTAATAA 380

| | | | ||| | | | | || | |

2147 CAGTATGTGCAGAATAATCCTCCCCCCTTGTTATTTTATCCCCGTTGTGA 2196

. . . . .

381 CACAC**AGGTGGTAG**CATCGCGCTGAGGGAGGGACAGATTTGC....GT**TA** 426

|| | | | | | | ||| | | | |||

2197 CATTTGC**ATTGACACCT**TTCCATTAG**GCCAGACTTTAA**CCTCCCCGCT**TA** 2246

. . . . .

427 **AT**ATCTCACTC....**GTCCTCTCGCACCT**TACCCAGCGCAGGC**CCCGCCC** 472

|| |||| | | || || ||||| | ||| |||||||||||

2247 **AT**CTCTCCCA**GTCC**CAACCC**TTCTCACCT**CCCTC**AGGTGAGG**C**CCCGCC**C 2296

GC box

*SmaI* *NheI*

473 **CGGG**C...GCTG**ATTGG**CTGGAGTGTTAGT**TATA**GTGACCCG**GCTAGC**GC 519

||||| ||||||||||||| | ||||||||| | || | |||

2297 CGGGCGCTGCTG**ATTGG**CTGGGGCAGTAGT**TATA**GCGGCCAAGGTAGATG 2346

CAAT box

TATA box

Motifs:

Homeodomain core

SMAD

T-box

Unknown

Sox -like

520 TGAAGTG**CAGA**G.**CAGA**GAG**A**

| |||||| || |

2347 CTCGGGG**CAGA**GT**CAAA**TTGAA**G**

Initiation
