# Supplementary figures and images for: Regulation of the Xenopus Xsox17α1 promoter by co-operating VegT and Sox17 sites
Source: Dev Biol. 2007 Oct 15;310(2):402–15. doi: 10.1016/j.ydbio.2007.07.028 (PMC2098691; doi:10.1016/j.ydbio.2007.07.028)

## Slide 1
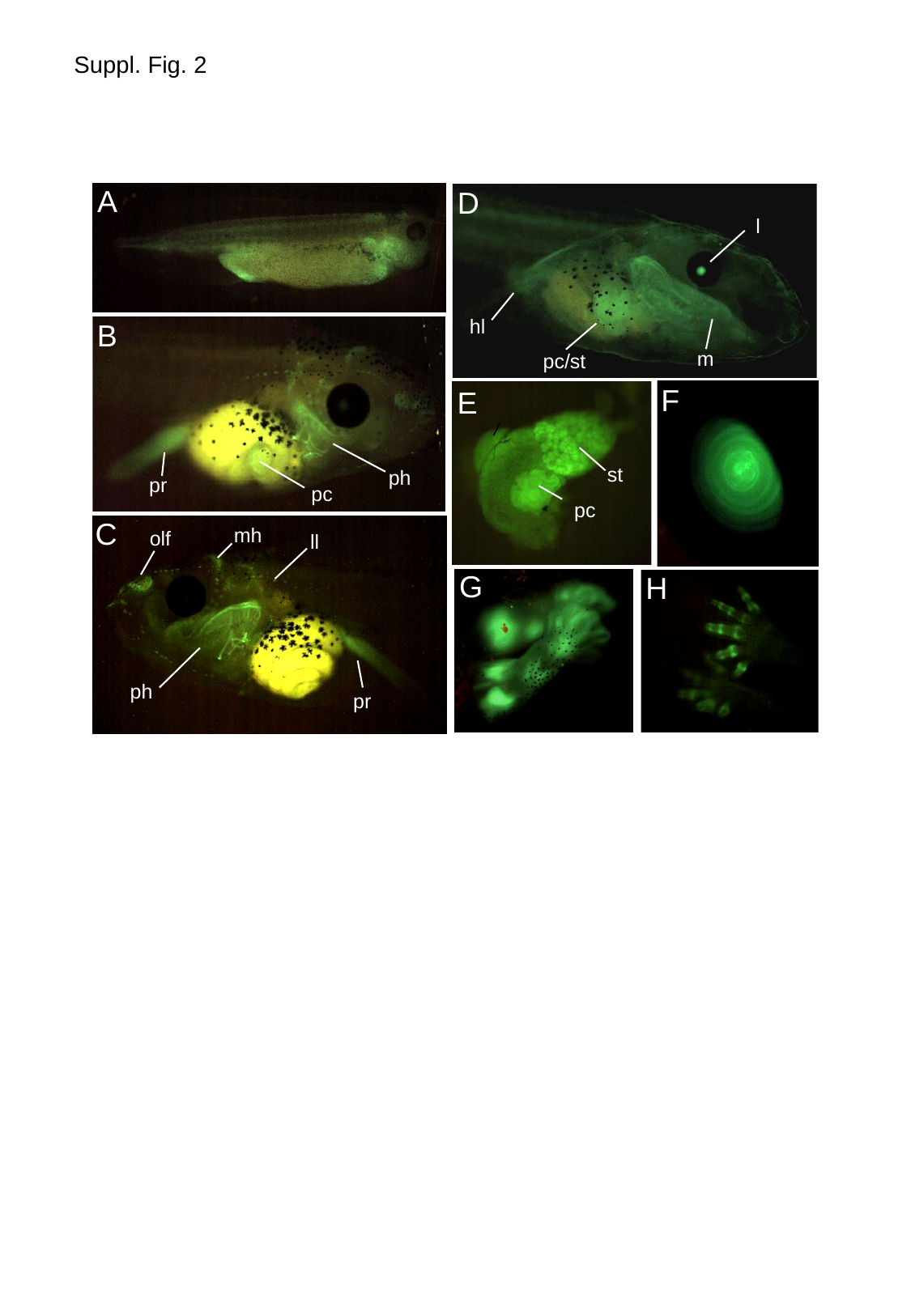

Suppl. Fig. 2
A
D
l
hl
B
m
pc/st
F
E
st
ph
pr
pc
pc
C
mh
olf
ll
G
H
ph
pr

Supplement: Supplementary Fig. 2 — Later expression of Xsox17α1 transgenes. Constructs were MR19 (A–C) and N-10 (D–H): (A) stage 37; (B–C) stage 47; (D–G) stage 54; (H) stage 56. (E–F) Regions dissected out for photography: (E) stomach/duodenum/pancreas, (F) lens. (G–H) Hind legs. Note the strong yellow fluorescence of the yolky gut. Abbreviations: hl, hind legs; l, lens; ph, pharynx; pc, pancreas; pr, proctodeum; ll, lateral line; mh, mid-hind brain boundary; olf, olfactory organ; m, mouth. [file mmc2.ppt]
