# Supplementary material for: Regulation of the Xenopus Xsox17α1 promoter by co-operating VegT and Sox17 sites
Source: Dev Biol. 2007 Oct 15;310(2):402–15. doi: 10.1016/j.ydbio.2007.07.028 (PMC2098691; doi:10.1016/j.ydbio.2007.07.028)
Supplement: Supplementary Fig. 3 — Sequences of endodermal sub-elements B1 and C3. Possible Smad sites (italics), FoxH1 (synonym Fast1) sites (blue), Sox binding sites (green) and T-box half site (bold underline). [file mmc3.ppt]

## Slide 1
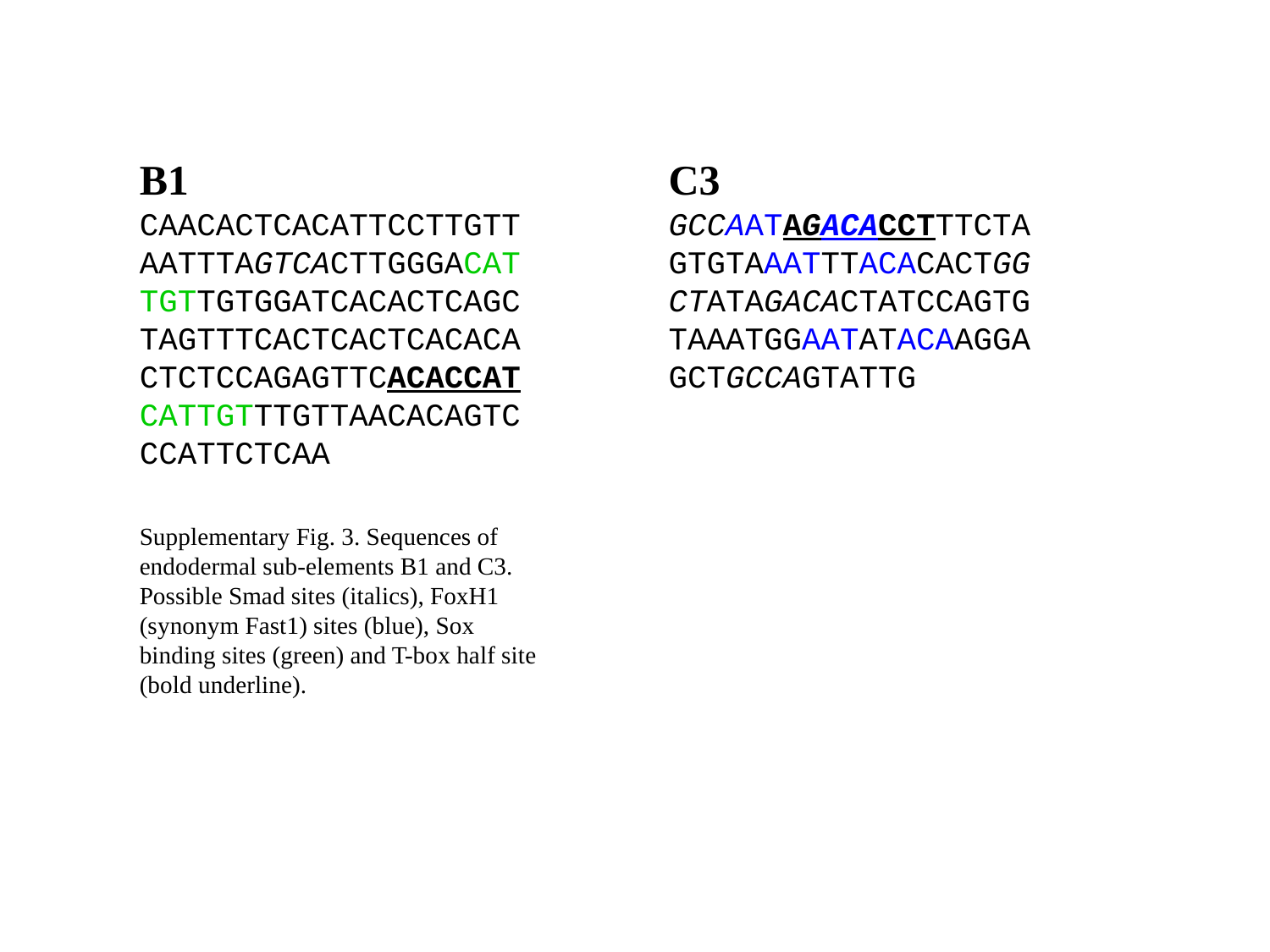

B1
CAACACTCACATTCCTTGTTAATTTAGTCACTTGGGACATTGTTGTGGATCACACTCAGCTAGTTTCACTCACTCACACACTCTCCAGAGTTCACACCATCATTGTTTGTTAACACAGTCCCATTCTCAA
Supplementary Fig. 3. Sequences of endodermal sub-elements B1 and C3.
Possible Smad sites (italics), FoxH1 (synonym Fast1) sites (blue), Sox binding sites (green) and T-box half site (bold underline).
C3
GCCAATAGACACCTTTCTAGTGTAAATTTACACACTGGCTATAGACACTATCCAGTGTAAATGGAATATACAAGGAGCTGCCAGTATTG
