# Supplementary material for: Regulation of the Xenopus Xsox17α1 promoter by co-operating VegT and Sox17 sites
Source: Dev Biol. 2007 Oct 15;310(2):402–15. doi: 10.1016/j.ydbio.2007.07.028 (PMC2098691; doi:10.1016/j.ydbio.2007.07.028)
Supplement: Supplementary Table 1 — Typical transgenic experiment analyzing deletion mutants. [file mmc4.doc]

Supplementary Table 1. Typical transgenic experiment analyzing deletion mutants.

| Construct | Number eggs injected | Number developing to gastrula | Number expressing GFP | % positives expressing in ectoderm | % positives expressing in endoderm |
| --- | --- | --- | --- | --- | --- |
| -12 | 219 | 41 | 19 | 100 | 100 |
| -12Δ10 | 193 | 34 | 13 | 100 | 0 |
| -12Δ10-5 | 262 | 53 | 23 | 100 | 0 |
| -10.5Δ7.5 | 209 | 36 | 20 | 100 | 100 |
| -10Δ7.5-5 | 239 | 54 | 25 | 100 | 100 |
| -10Δ5 | 252 | 51 | 5 | 100 | 100 |
| -1.7 | 135 | 27 | 5 | 100 | 0 |

The structures of the constructs are shown in Fig. 2.
