# Supplementary material for: Regulation of the Xenopus Xsox17α1 promoter by co-operating VegT and Sox17 sites
Source: Dev Biol. 2007 Oct 15;310(2):402–15. doi: 10.1016/j.ydbio.2007.07.028 (PMC2098691; doi:10.1016/j.ydbio.2007.07.028)
Supplement: Supplementary Table 2 — Analysis of normal transgenic mid-gastrula (stage 10.5) embryos and transgene expression in experiments analyzing the effects of endodermal element mutations. [file mmc5.doc]

Supplementary Table 2. Analysis of normal transgenic mid-gastrula (stage 10.5) embryos and
transgene expression in experiments analyzing the effects endodermal element mutations.

| Construct | Number of embryos cleaving normally and transgenic* | % Endodermal expression | % Weak endodermal expression | % Endoderm negative | % Enhanced animal expression |
| --- | --- | --- | --- | --- | --- |
| B1 | 53 | 60 | 0 | 0 | 0 |
| C3 | 43 | 65 | 0 | 0 | 0 |
| B1 Tbox Mutant | 46 | 0 | 63 | 0 | 0 |
| B1 Sox Mutant | 28 | 0 | 64 | 0 | 46 |
| B1 Triple Mutant | 28 | 0 | 0 | 75 | 0 |

*Some transgenic embryos showed weak or very mosaic expression and therefore could not be
scored according to the criteria in later columns.
